# Pro-inflammatory cytokines induce cell death, inflammatory responses, and endoplasmic reticulum stress in human iPSC-derived beta cells

**DOI:** 10.1186/s13287-019-1523-3

**Published:** 2020-01-03

**Authors:** Stéphane Demine, Andrea Alex Schiavo, Sandra Marín-Cañas, Piero Marchetti, Miriam Cnop, Decio L. Eizirik

**Affiliations:** 1 ULB Center for Diabetes Research, Medical Faculty, Université Libre de Bruxelles (ULB), Route de Lennik 808-CP618, 1070 Brussels, Belgium; 2 Indiana Biosciences Research Institute, Indianapolis, IN USA; 3 Department of Clinical and Experimental Medicine, University of Pisa, Pisa, Italy; 4 Division of Endocrinology, Erasmus Hospital, Université Libre de Bruxelles, 1070 Brussels, Belgium

**Keywords:** Pancreatic beta cells, Type 1 diabetes, Induced pluripotent stem cells, Cytokines, Apoptosis, Endoplasmic reticulum stress

## Abstract

**Background:**

Adult human pancreatic beta cells are the “gold standard” for studies on diabetes pathogenesis, but their use is limited by insufficient availability and variable quality. An important effort has recently taken place to differentiate beta cells from human induced pluripotent stem cells (iPSCs) and validate their use for diabetes research. We presently used a 7-stage protocol to generate beta cells from human iPSC and evaluated whether these cells are responsive to the pro-inflammatory cytokines (IFNγ, IL-1β, or IFNα) that play a role in type 1 diabetes.

**Methods:**

The iPSC-derived islet-like cell clusters contained 40–50% beta and 10–15% alpha cells and expressed the receptors for IFNγ, IL-1β, or IFNα. Cells were exposed to either IFNγ (1000 U/mL) + IL-1β (50 U/mL) or IFNα alone (2000 U/mL) for 24/48 h. Apoptosis was quantified using Hoechst/propidium iodide staining or the RealTime Glo Apoptosis Kit (Promega). After treatment, CXCL10 secretion was quantified by ELISA. The expression of multiples genes (*Ins*, *Gcg*, *Nkx2.2*, *Nkx6.1*, *Pdx1*, *Mafa*, *BiP*, *Chop*, *Atf3*, *CXCL10*, *CXCL9*, *CCL5*, and *HLA-ABC*) was quantified by RT-qPCR. Phosphorylation state and total expression of STAT1/STAT2, as well as expression of PDL1 and of the ER chaperone BiP, were quantified by Western blotting. The co-localization of HLA-ABC or cleaved caspase-3 and Ins/Gcg expression was assessed by immunohistochemistry. The presence of HLA-ABC at the plasma membrane was measured by flow cytometry.

**Results:**

IFNγ + IL-1β and IFNα induced apoptosis of the cells after 48 h of exposure. Cleaved caspase-3 co-localized mostly but not exclusively with Ins+ cells. Exposure to IFNγ + IL-1β induced a pro-inflammatory phenotype, including increased *CXCL10*, *CXCL9*, and *CCL5* expression; CXCL10 secretion; and *HLA-ABC* expression. HLA overexpression was confirmed at the protein level by Western blotting and flow cytometry. Exposure to IFNγ + IL-1β (but not IFNα) also induced beta cell dedifferentiation and endoplasmic reticulum stress (increase in *BiP*, *Chop*, and *Atf3* mRNA expression). Phosphorylation of STAT1 was stimulated already after 1 h by IFNγ + IL-1β and IFNα, while phosphorylation of STAT2 was only activated by IFNα at 1–4 h. PDL1 expression was increased by both IFNγ + IL-1β and IFNα.

**Conclusions:**

Our data show that human iPSC-derived beta cells respond to pro-inflammatory cytokines IL-1β + IFNγ and IFNα, by activating the same pathogenic processes as adult human primary beta cells. These cells thus represent a valuable tool for future research on the pathogenesis of type 1 diabetes.

## Background

Type 1 diabetes (T1D) is characterized by an autoimmune attack targeting specifically the beta cells [1], but the molecular mechanisms underlying this disease are not yet fully understood. None of the currently available treatments modifies the progressive nature of T1D, eventually leading to a near complete loss of endogenous insulin secretion in these patients. Important species differences between rodent models and human disease [2] highlight the need for research on human islets. A major roadblock is the difficulty to obtain human islets: they are only available in a few transplantation centers worldwide and depend on scarce organ donation.

A decade ago, the technology to induce pluripotent stem cells (iPSCs) was applied for the first time to human cells [3]. This method redefined the stem cell field and opened the possibility to study diseases and screen drugs in vitro in a patient-specific manner [4, 5]. In 2014, two teams published methods to generate glucose-responsive beta cells from iPSCs [6, 7]. So far, iPSC-derived beta cells have been mostly used to study pathogenic mechanisms underlying different monogenic forms of diabetes, i.e., patients with neonatal diabetes [8, 9], mature-onset diabetes of the young [10], Wolfram syndrome [11], and TRMT10A deficiency [12]. There have also been attempts to generate stem-cell-derived beta cells from patients with T1D [13] or from a fulminant form of severe-insulin-dependent diabetes prevalent in Japan [14]. These cells have so far not been fully validated, however, as a model to study the mediators of beta cell death in T1D, particularly regarding the susceptibility of iPSC-derived beta cells to the pro-inflammatory cytokines IL-1β, IFNγ, and IFNα. These cytokines contribute to beta cell dysfunction and death in T1D via induction of endoplasmic reticulum (ER) stress [15, 16], HLA class I (HLA-ABC) upregulation [17], chemokine production, and apoptosis [1, 17].

We presently evaluated whether beta cells differentiated from iPSC lines [12] are responsive to pro-inflammatory cytokines. The data show that human iPSC-derived beta cells respond to the pro-inflammatory cytokines IL-1β + IFNγ and IFNα, similarly to adult human primary beta cells. They thus provide a useful model to better understand the pathogenesis of T1D and screen for new drugs aiming to protect beta cells in early disease.

## Material and methods

### Cell origin, ethical information, and differentiation of iPSCs into beta cells

Fibroblasts from human neonatal foreskin [18] and umbilical cord [12] were obtained from healthy donors after informed consent, with approval by the Ethics Committees of the Helsinki and Uusimaa Hospital District (Helsinki, Finland) and the Erasmus Hospital (ULB, Brussels, Belgium). These cells were reprogrammed into iPSCs as previously described [8], leading to the generation of two independent control iPSC lines, namely HEL46.11 [18] and HEL115.6 [12]. The full characterization of these iPSC lines is available in previous publications [12, 18]. iPSCs were differentiated into beta cells using a 7-step protocol previously published by our group [12]. At the end of the stage 4, the cells were seeded in 24-well microwell plates at a density of 9.10^5^ cells per well (Aggrewell 400, Stem Cell Technologies, Vancouver, Canada) and the differentiation was continued in these plates according to the protocol previously published by our group [12].

### Cell exposure to cytokines

At the end of the differentiation, cell aggregates were resuspended from microwell plates, transferred into 10-cm Petri dishes, and manually collected using a micropipette (200 aggregates per condition). The retrieved aggregates were washed twice with pre-warmed PBS and resuspended in 1 mL of HAM’s F-10 nutrient mixture (Thermofisher, Waltham, MA, USA) containing 3.75 g free fatty acid-free BSA (bovine serum albumin) (Roche, Basel, Switzerland), 2.5 mL GlutaMAX (Thermofisher), and 100 U/mL penicillin-streptomycin (Thermofisher), and cytokines were added (1000 U/mL IFNγ (Peprotech, London, UK) + 50 U/mL IL-1β (R&D Systems, Abingdon, UK) [16] or 2000 U/mL IFNα (Peprotech) [17]). Cells were exposed to cytokines for 24 or 48 h, as described in figure legends.

### Apoptosis assays

Cell death was determined on whole cell aggregates using Hoechst 33342 (Sigma, Saint-Louis, MI, USA) and propidium iodide staining (Sigma) and fluorescence microscopy, as described previously [19]. Apoptosis was determined by two researchers, one of them unaware of the experimental conditions tested. Apoptosis and necrosis were also determined using an annexin V-based fluorescent assay (RealTime-Glo™ MT Cell Viability Assay, Promega, Madison, WI, USA), according to the manufacturer’s instructions. Fluorescence and luminescence were recorded after 0, 4, 8, 16, 24, and 48 h of incubation using a VictorX5 multilabel plate reader (Perkin-Elmer, Waltham, MA, USA). Results were expressed as arbitrary fluorescence units (AFU; necrosis/plasma membrane integrity) or arbitrary luminescence units (ALU; apoptosis) and calculated as fold change to time 0.

### mRNA extraction and RT-qPCR

Messenger RNA was isolated from cell aggregates using the Dynabeads mRNA DIRECT Purification Kit (Thermofisher), according to the manufacturer’s instructions. RNA was retrieved in Tris HCl solution and reverse transcription done using the Reverse Transcriptase Core kit (Eurogentec, Liège, Belgium), according to the manufacturer’s instructions. Gene expression was assessed using real-time PCR (Rotor Gene Q machine, Qiagen, Hilden, Germany) with the primers described in Additional file 1: Table S1. Gene expression was corrected for the reference gene beta-actin and data are expressed as fold change of untreated cells.

### Western blotting

Cell aggregates were resuspended in RIPA (radioimmunoprecipitation assay) buffer (Sigma) containing cOmplete Ultra Protease Inhibitor Cocktail (Roche), sonicated (3 × 10 s) on ice to ensure complete lysis (Bioruptor Plus, Diagenode, Liège, Belgium), and centrifuged for 13,000*g* for 10 min at 4 °C to remove debris and undigested cells. Protein concentration was quantified using a BCA protein assay kit (Thermofisher). Fifty-microgram protein was loaded on a 10–12% SDS-PAGE gel. Samples were transferred to a nitrocellulose membrane and detected using primary antibodies listed in Additional file 1: Table S2.

### Immunofluorescence

Cells were washed twice with PBS containing 1 mM EDTA and incubated in 1 mL Accutase (Stemcell Technologies, Vancouver, Canada) for 5 min at 37 °C with mild agitation. Reaction was stopped by adding 10% Knock-Out Serum (Thermofisher). Cells were centrifuged at 700*g* for 5 min at room temperature and resuspended in 1 mL HAM’s F-10 medium, supplemented as indicated above. Seventy thousand cells in a 500-μL volume medium were seeded per square ICC chamber (Nunc Lab-Tek II, Thermofisher). After 24 h, cells were exposed to pro-inflammatory cytokines as described above. Cells were fixed for 15 min at room temperature with 4% paraformaldehyde, permeabilized for 30 min with 0.1% PBS–Triton X100, and blocked for 8 min with Ultravision protein block (Thermofisher), using antibodies and incubation conditions described in Additional file 1: Table S2. Finally, cells were mounted using Vectashield Vibrance Antifade Mounting Medium (Vector Laboratories, Peterborough, UK). Pictures were taken using a fluorescence microscope (Axiovert, Zeiss, Oberkochen, Germany).

### Confocal microscopy

The staining procedure was carried out in suspension in 1.5-mL microcentrifuge tubes (centrifugation steps were performed at 300*g* for 5 min). Aggregates were collected and washed twice in PBS; fixation was carried out with 4% paraformaldehyde for 1 h at room temperature. Samples were permeabilized for 30 min in 0.5% Triton X-100 in PBS. After one wash, blocking of non-specific binding was performed by adding Ultravision Protein Block for 15 min. Antibodies and incubation conditions are described in Additional file 1: Table S2. Nucleus counterstaining was performed using SYTOX Blue (Thermofisher). Samples were resuspended in Glycergel Mounting Medium (Agilent/Dako, Santa Clara, CA, USA), transferred to a slide, and covered with a glass coverslip. Imaging was performed using an Inverted Zeiss LSM 510 confocal microscope (Zeiss). Co-localization between different signals was assessed using Imaris software (Oxford Instruments, Abingdon-on-Thames, UK) and built-in co-localization analysis function.

### CXCL10 secretion quantification

Secreted CXCL10 was quantified in culture media using anti-human CXCL10 ELISA according to the manufacturer’s instructions (R&D Systems). Results were normalized for total protein content of the aggregates, quantified by the BCA method.

### Flow cytometry

Cell aggregates were dissociated as described in the “immunofluorescence” section. 10^6^ living cells were incubated in ice-cold PBS containing BSA 0.5%, 2 mM EDTA, and conjugated antibody targeting HLA-ABC. Viability was assessed by using Zombie Aqua (Biolegend, San Diego, CA, USA). After two washes, cells were fixed and permeabilized using Cytofix/Cytoperm Kit (BD Biosciences Erembodegem, Belgium) according to the manufacturer’s instructions. Cells were finally stained for Ins and Gcg. Antibodies and incubation conditions are described in Additional file 1: Table S2. Cells were kept on ice during the procedure to prevent HLA-ABC internalization and then analyzed using a BD LSR Fortess X-20 (BD Biosciences) using proper isotype controls for gating. A first gate was used to select the cells negative for Zombie Aqua signal (viable cells). Four cell populations were gated based on the Ins and the Gcg signal. The number of HLA-positive cells was assessed in each cell population. Data analysis was carried out with FlowJo software (Version 10, FlowJo, Ashland, OR, USA).

### Statistical analysis

Data are presented as means ± SEM and/or plotted as scatter plots. Analyses were performed by paired or unpaired one-way ANOVA followed by Student’s *t* test or by paired or unpaired Student’s *t* test with Bonferroni’s correction for multiple comparisons, using Graph Pad Prism 7 software (GraphPad Software, La Jolla, CA, USA). A *p* value ≤ 0.05 was considered as significant.

## Results

**Fig. 1:**
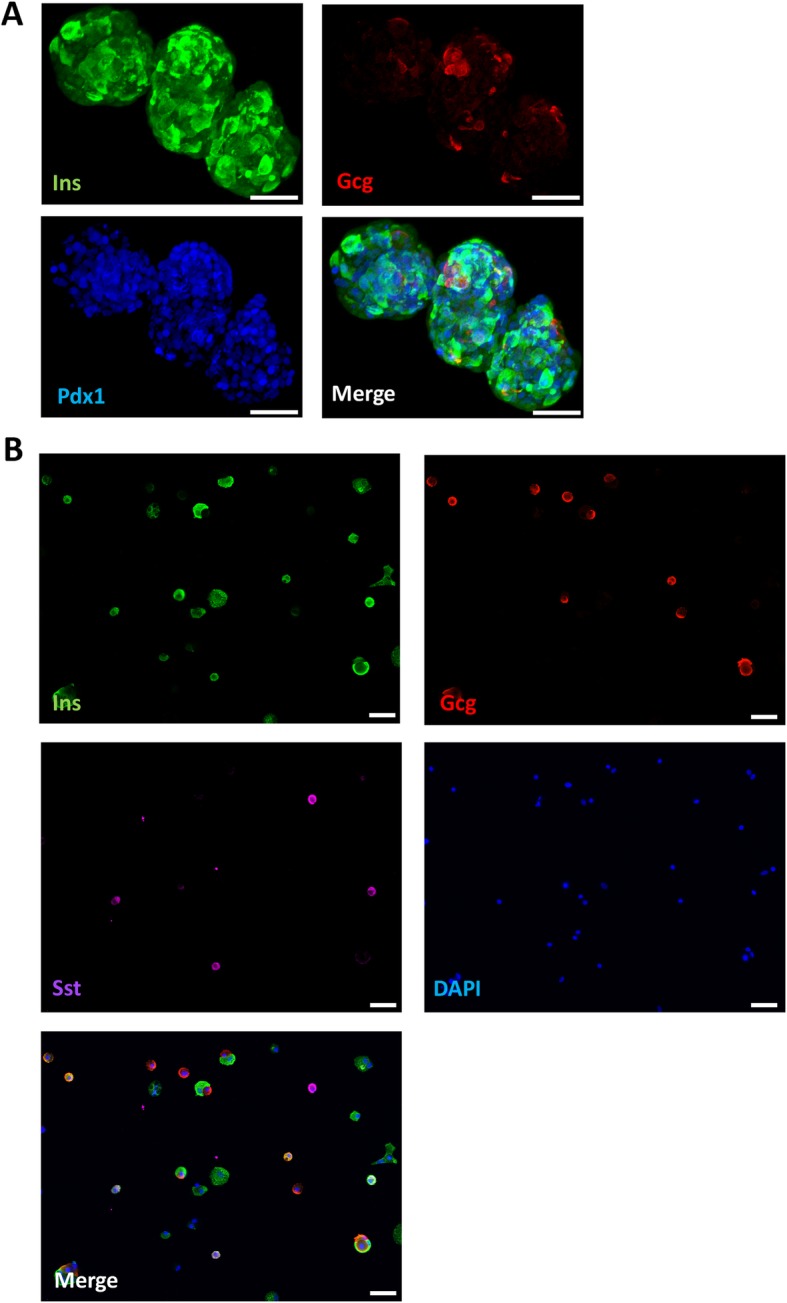
Differentiation of control iPSCs into pancreatic endocrine cells. Control iPSCs (HEL115.6) were differentiated into pancreatic endocrine cells using a 7-step protocol. At the end of stage 7, a mixed cell population comprised of insulin- (Ins), glucagon- (Gcg), somatostatin-positive cells (Sst), and polyhormonal and non-hormonal cells was obtained. Whole aggregates (**a**) or dispersed aggregates (**b**) were fixed and expression of insulin, somatostatin and glucagon was assessed (Ins green, Gcg red, Sst purple). Nuclei were counterstained with Pdx1 immunostaining (**a**) or DAPI (blue, **b**). Pictures were taken with an epifluorescence microscope with × 20 (scale bar = 50 μm) magnification

Pancreatic endocrine cells were differentiated from control iPSC lines as described before [12]. This 7-stage differentiation protocol uses monolayer culture up to pancreatic endoderm (stage 4) and then three-dimensional culture enabling the formation of islet-like aggregates [12]. At the end of differentiation, the aggregates contained insulin-, glucagon- (Fig. 1a, b), and somatostatin-expressing (Fig. 1b) cells, as well as polyhormonal and non-endocrine cells (Fig. 1a, b) [12]. Each preparation was characterized by RT-qPCR across stages of differentiation (Additional file 1: Figure S1). To allow a more precise characterization of the different cells obtained, aggregates were dispersed and seeded on Matrigel-coated culture plates (Fig. 1b). Beta cells accounted for 43 ± 17% of the cells, alpha cells represented 8 ± 5%, and some polyhormonal cells, i.e., positive for both insulin and glucagon, were also present (5 ± 3%, mean ± SEM, *n* = 10).

As the first step to assess the responsiveness of these cells to the pro-inflammatory cytokines IFNγ, IL-1β, and IFNα, we determined the expression of genes encoding receptors for these cytokines in iPSC-derived beta cells as compared to primary human islets (4 human islet preparations, obtained as previously reported [20] and described in Additional file 1: Methods and Additional file 1: Table S3). The receptors *IFNGR1* (for IFNγ), *IL1R1* (for IL-1β), and *IFNAR1* (for IFNα) were indeed present at stage 7, at levels comparable to those in primary human islets (Additional file 1: Figure S2).

**Fig. 2:**
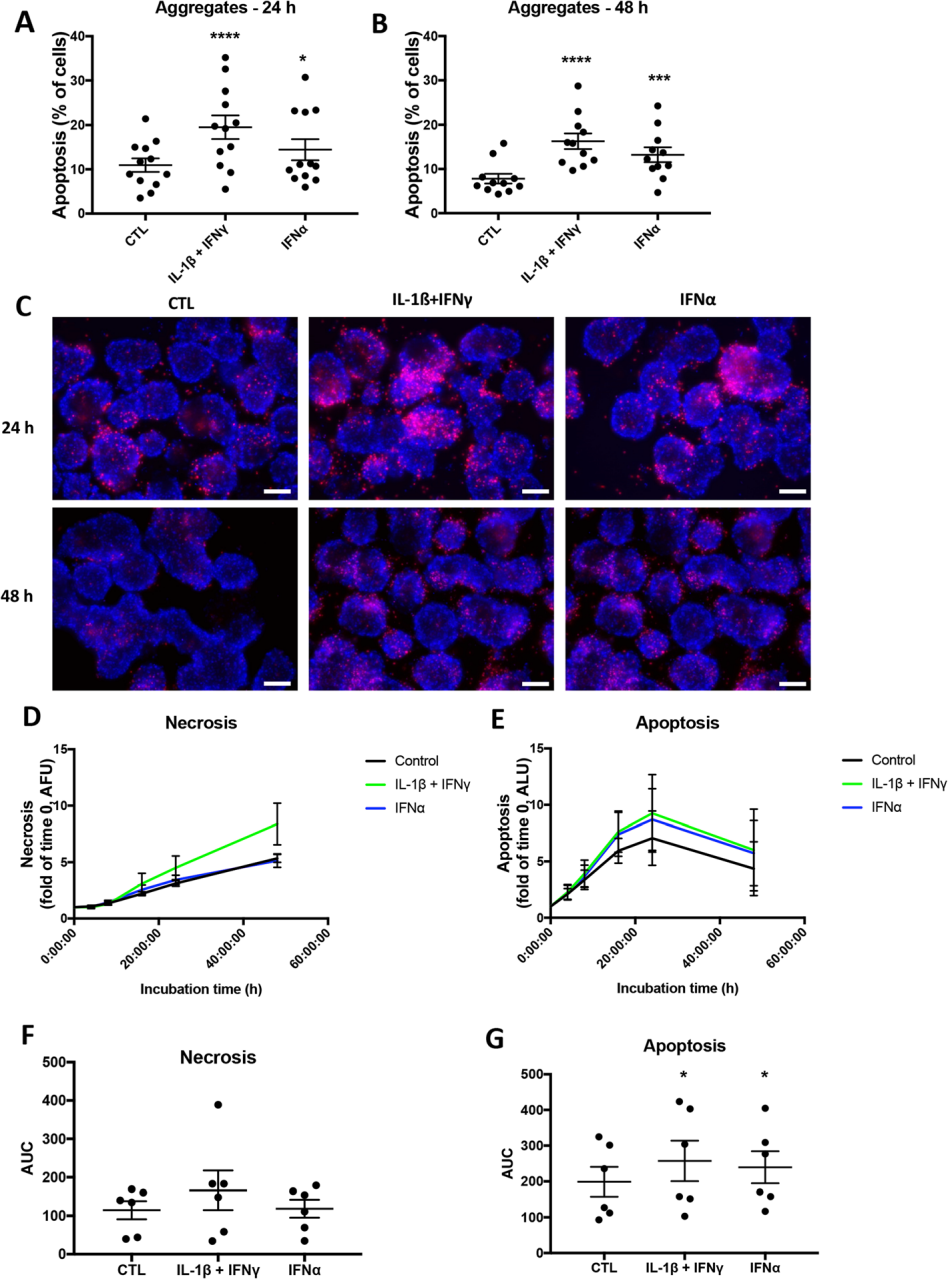
The pro-inflammatory cytokines IFNγ + IL-1β and IFNα induce apoptosis in iPSC-derived pancreatic endocrine cells. Control iPSCs (HEL115.6) were differentiated into endocrine pancreatic cells. The islet-like aggregates were exposed to IFNγ (1000 U/mL) + IL-1β (50 U/mL) or to IFNα (2000 U/mL) for 24 or 48 h. **a**, **b** Apoptosis was counted following Hoechst 33342 and propidium iodide staining (*n* = 12 independent experiments). **c** Representative pictures taken with an epifluorescence microscope with × 20 magnification (scale bar = 100 μm). **d**, **e** Aggregates were mixed with RealTime Glo solution and exposed to cytokines. Fluorescence and luminescence were recorded after 0, 4, 8, 16, 24, and 48 h. Results are expressed as arbitrary fluorescence units (AFU; necrosis/plasma membrane integrity) or arbitrary luminescence units (ALU; apoptosis), calculated as fold change of the value obtained at time 0 (control) (*n* = 6 independent experiments). **f**, **g** For each experiment, the area under the curve (AUC) was calculated. CTL denotes control. *p ≤ 0.05, ***p ≤ 0.001, ****p ≤ 0.0001 (paired Student’s *t* test)

The iPSC-derived aggregates were exposed to IFNγ + IL-1β or to IFNα alone. IFNγ + IL-1β increased apoptosis by around twofold after 24 and 48 h of culture (Fig. 2a–c). IFNα induced less marked cell death (1.3–1.6-fold vs untreated cells) after 24 and 48 h (Fig. 2a–c). No increase in necrosis was observed under these experimental conditions (data not shown). Similar results were obtained using aggregates from another control iPSC line, HEL46.11 (Additional file 1: Figure S3). In dispersed primary human islets, IFNγ + IL-1β but not IFNα alone induced apoptosis (Additional file 1: Figures S4A and S5A).

Cell death was also quantified using the RealTime Glo kit, which allows to simultaneously quantify necrosis by measuring plasma membrane integrity and apoptosis by detecting phosphatidylserine flip-flop using Annexin V conjugates. There was no significant cytokine-induced necrosis (Fig. 2d, f), but annexin V staining increased for cells exposed to IFNγ + IL-1β or IFNα alone (Fig. 2e, g). Altogether, these data suggest that iPSC-derived pancreatic endocrine cells are susceptible to cytokine-induced cell death.

**Fig. 3:**
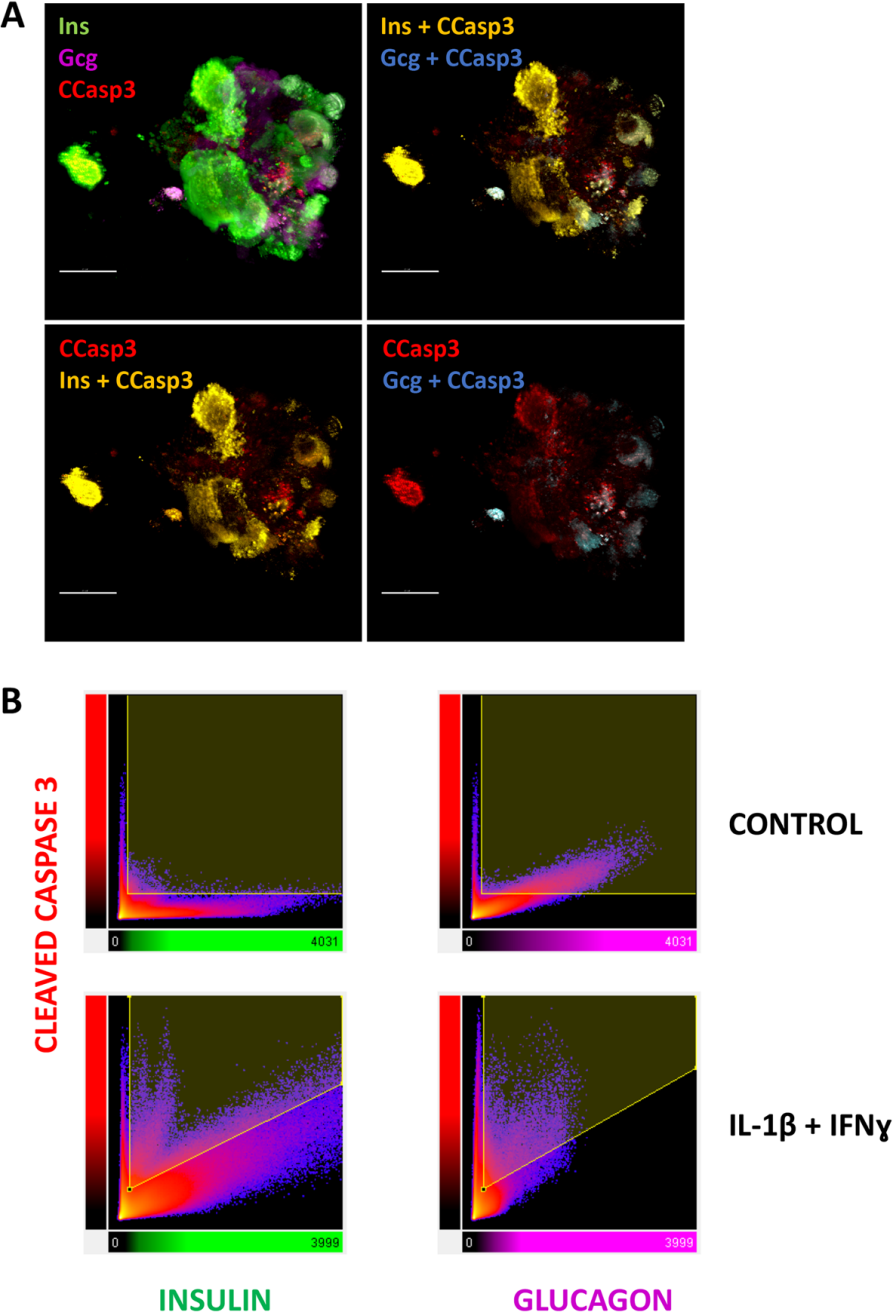
Co-localization of cleaved caspase 3, insulin, and glucagon in iPSC-derived pancreatic endocrine cells exposed to IFNγ + IL-1β. Control iPSCs (HEL115.6) were differentiated into pancreatic endocrine cells and exposed to IFNγ + IL-1β for 24 h. **a** Cells were fixed, and expression of cleaved caspase 3 (CCasp3), insulin (Ins), and glucagon (Gcg) was assessed. Nuclei were counterstained with SYTOX Blue. Pictures were taken with a confocal microscope. Co-localization of different signals (indicated in the pictures) was analyzed using pseudo colors indicating co-localization. **b** Co-localization between cleaved caspase 3, insulin, and glucagon was assessed using Imaris software and built-in co-localization analysis function. Data are presented as plots of cleaved caspase 3 signal and insulin or glucagon signal in control or cytokine-exposed cells

To identify which cell type undergo cell death, cleavage of caspase 3 was examined by immunocytochemistry on aggregates exposed to IFNγ + IL-1β for 24 h. Insulin, glucagon, and polyhormonal and non-endocrine cells were positive for cleaved caspase 3 (Fig. 3a). Quantification of the co-localization with hormones confirmed that caspase 3 cleavage occurred in both beta and alpha cells (Fig. 3b), with a stronger correlation between cleaved caspase 3 and insulin compared to glucagon, suggesting that beta cells were preferentially affected by the cytokines (Fig. 3b).

**Fig. 4:**
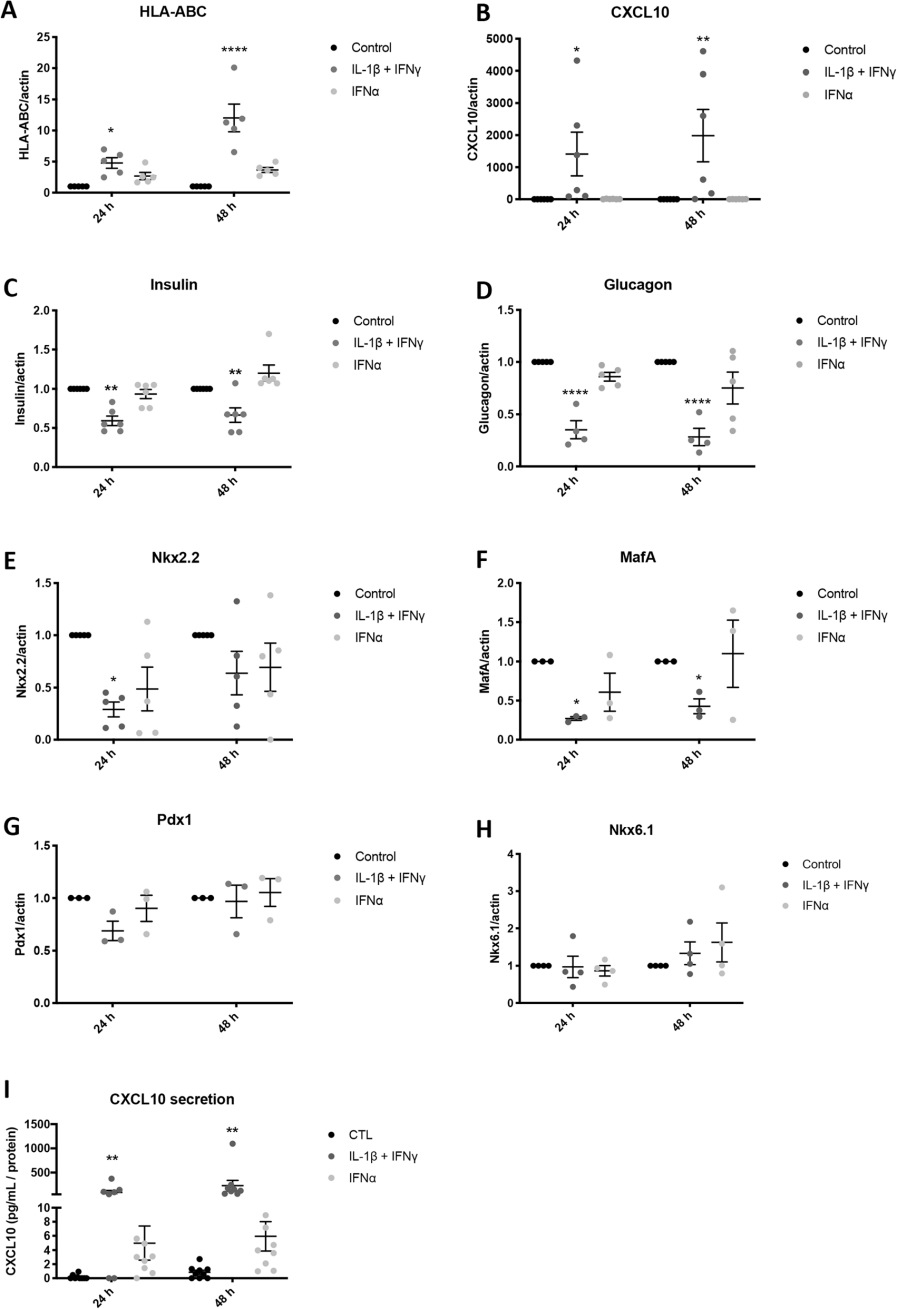
IFNγ + IL-1β and IFNα induce gene expression changes in iPSC-derived pancreatic endocrine cells. Control iPSCs (HEL115.6) were differentiated into pancreatic endocrine cells and exposed to IFNγ + IL-1β or IFNα for 24 and 48 h. **a**–**h** The expression of *HLA-ABC*, *Cxcl10*, *Ins*, *Gcg*, *Nkx2-2*, *MafA*, *Pdx1*, *Nkx6-1*, and *Actin* (reference gene) was quantified by RT-qPCR. Expression was corrected for the corresponding actin value and expressed as fold change compared to untreated cells (control) (*n* = 3–6 independent experiments). **i** CXCL10 secretion to the culture medium was quantified by ELISA. Data were calculated as pg/mL CXCL10 in the medium and normalized for total protein content (*n* = 8 independent experiments). *p ≤ 0.05, **p ≤ 0.01, ****p ≤ 0.0001 (one-way ANOVA followed by paired Student’s *t* test)

We next evaluated whether cytokine exposure of iPSC-derived aggregates led to expression of inflammation-related genes or affected genes related to the endocrine cell phenotype (Fig. 4). IFNγ + IL-1β strongly induced *HLA-ABC* (human leukocyte antigen) and *CXCL10* (C-X-C motif chemokine 10) mRNA expression at 24 and 48 h, while with IFNα there was only a trend for increased *HLA-ABC* mRNA expression and no increase in *CXCL10* (Fig. 4a, b). The results were similar to the effects of IFNγ + IL-1β on *HLA-ABC* and *CXCL10* expression in human islets (Additional file 1: Figure S5B-C; these data, re-calculated from our previous studies [17, 21], are shown here for comparison with the iPSC data). However, differently to iPSC-derived cells, an increase in *CXCL10* expression was found in human islets treated with IFNα alone (Additional file 1: Figure S4B-C). The increase in *CXCL10* mRNA expression in beta cells derived from iPSCs in response to IFNγ + IL-1β was confirmed at the protein level by ELISA, with augmented CXCL10 release into the medium (Fig. 4i). The mRNA expression of other chemokines, i.e., CCL5 (Chemokine (C-C motif) ligand 5) and CXCL9, was induced by IFNγ + IL-1β (Additional file 1: Figure S6 G-J).

**Fig. 5:**
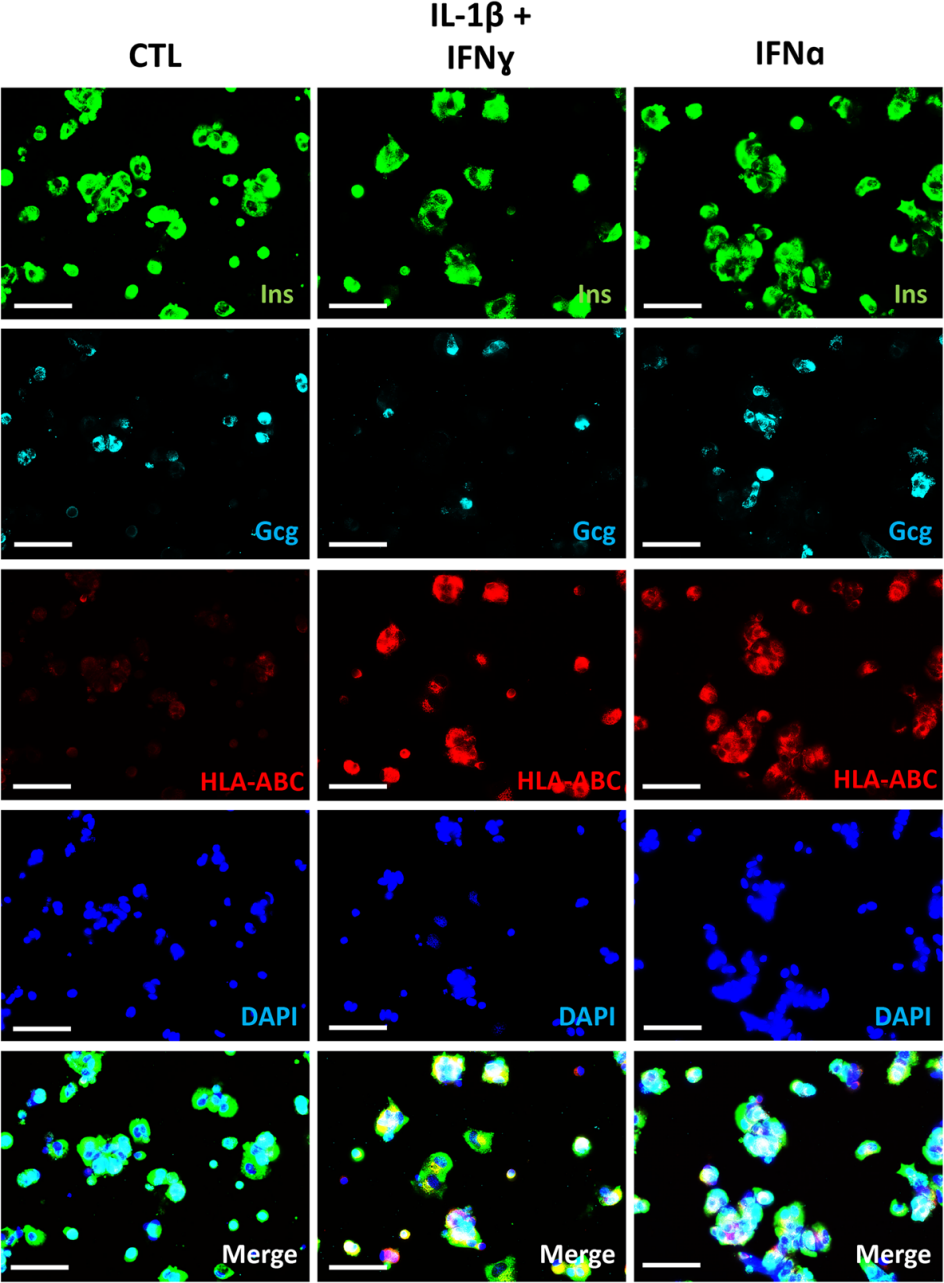
HLA-ABC expression in IFNγ + IL-1β- and IFNα-exposed iPSC-derived pancreatic endocrine cells. Control iPSCs (HEL115.6) were differentiated into pancreatic endocrine cells and exposed to IFNγ + IL-1β or IFNα. Cells were fixed, and expression of insulin (Ins), glucagon (Gcg) and HLA-ABC was evaluated (Ins green, HLA-ABC red, Gcg far red). Nuclei were counterstained with DAPI. Pictures were taken with a fluorescence microscope with × 20 magnification (scale bar = 50 μm) and are representative of six independent experiments

**Fig. 6:**
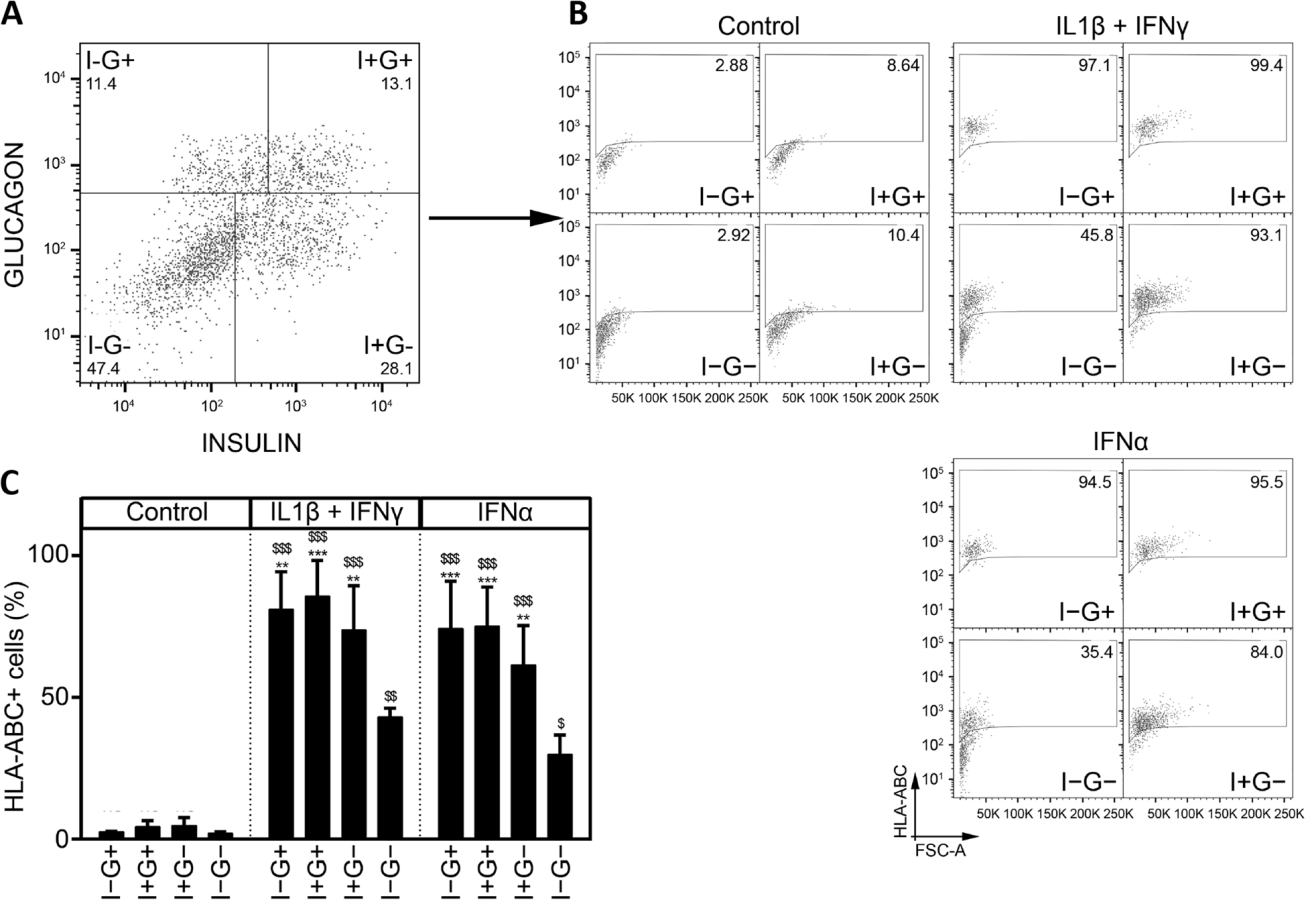
HLA-ABC is expressed at the plasma membrane of IFNγ + IL-1β- and IFNα-treated iPSC-derived pancreatic endocrine cells. Control iPSCs (HEL115.6) were differentiated into pancreatic endocrine cells and exposed to IFNγ + IL-1β or IFNα for 24 h. Cells were dissociated, and expression of insulin (Ins), glucagon (Gcg), and HLA-ABC was evaluated by flow cytometry. **a** Four cell populations were identified using Ins (indicated as I+) and Gcg (indicated as G+) expression (respectively I+G−, I−G+, I+G+, and I−G−). **b** For each cell population defined above, HLA-ABC expression was plotted against the forward scatter signal. **c** A histogram showing the percentages of cells expressing HLA-ABC at their surface, for each of the cell population identified (*n* = 4 independent experiments). **p ≤ 0.01, ***p ≤ 0.001 (one-way ANOVA followed by paired Student’s *t* test; significant compared to corresponding I−G− cell population); ^$^*p* ≤ 0.05, ^$$^*p* ≤ 0.01, ^$$$^*p* ≤ 0.0001 (one-way ANOVA followed by paired Student’s *t* test; significant compared to respective control condition)

To assess HLA-ABC protein expression, we co-stained dispersed iPSC-derived aggregates for insulin, glucagon, and HLA-ABC. IFNγ + IL-1β increased HLA-ABC expression in both alpha and beta cells (Fig. 5). The response to IFNα was less marked, with HLA-ABC induction in only three out of six experiments, at levels below those observed with IFNγ + IL-1β (data not shown). As HLA-ABC cellular localization is important for its function, HLA-ABC expression was further evaluated by flow cytometry. There was a significantly increased membrane expression of HLA-ABC in cells treated with IFNγ + IL-1β or IFNα (Fig. 6a–c). This increase in HLA-ABC membrane expression was present in all cell types but was significantly higher in insulin and glucagon-positive cells and polyhormonal cells (Fig. 6a–c), as compared to non-endocrine cells. The observed differences between ICC and flow cytometry results may be explained by the higher sensitivity of the later technique.

The expression of genes encoding pancreatic hormones (Ins, Gcg; Fig. 4c, d) and key transcription factors for the maintenance of the beta cell phenotype (Nkx2.2, MafA; Fig. 4e, f) were decreased in response to IFNγ + IL-1β but not to IFNα. Nkx6.1 and Pdx1 expression were not affected by cytokines (Fig. 4g, h). There were no significant changes in medium insulin accumulation in iPSC-derived cells treated with IFNγ + IL-1β or IFNα (Additional file 1: Figure S7).

**Fig. 7:**
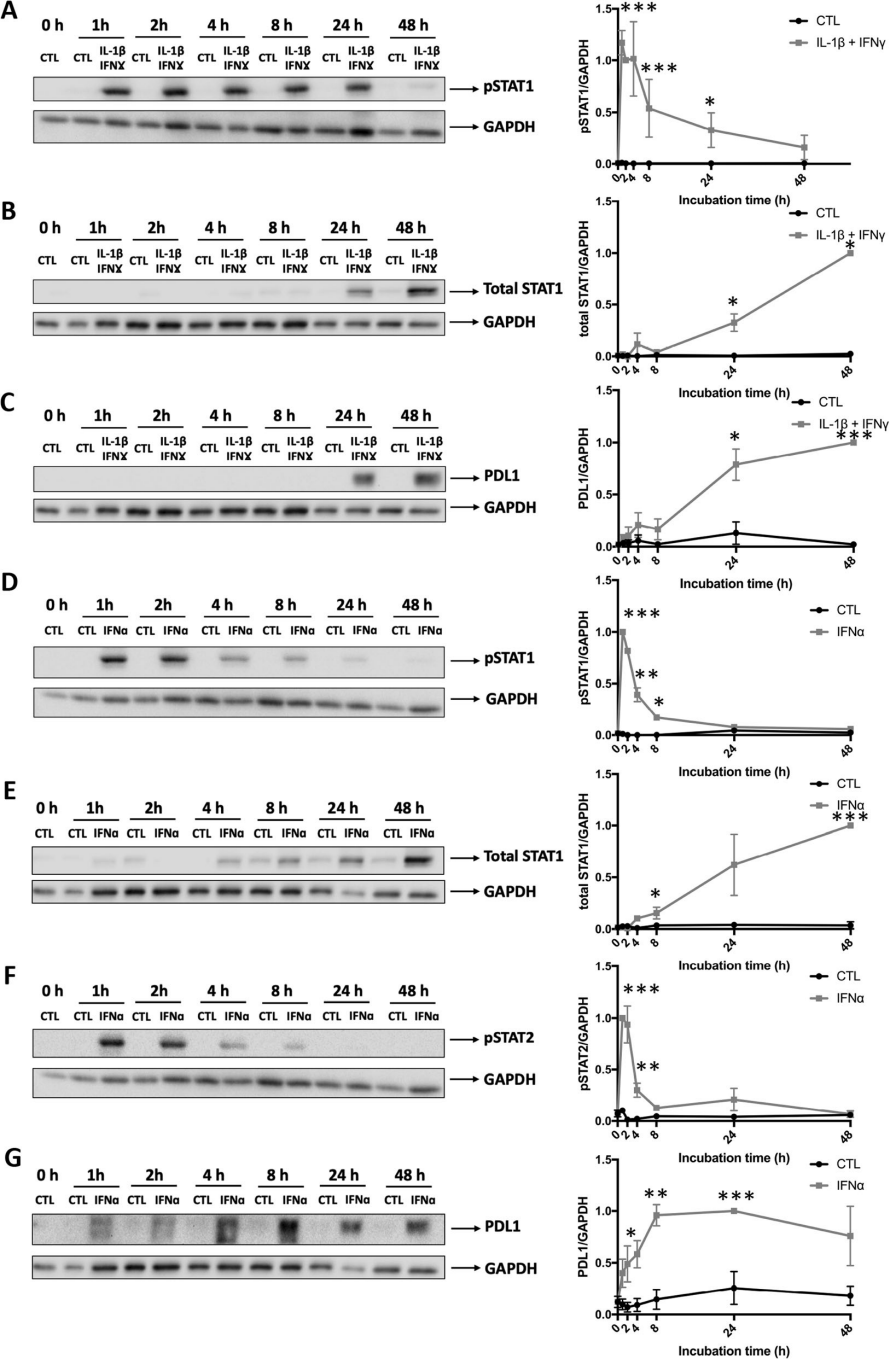
Time course analysis of IFNγ + IL-1β- or IFNα-induced STAT1, STAT2, and PDL1 expression in iPSC-derived pancreatic endocrine cells. Control iPSCs (HEL115.6) were differentiated into pancreatic endocrine cells and exposed to IFNγ + IL-1β (**a**–**c**) or IFNα (**d**–**g**) for 0, 1, 2, 4, 8, 24, and 48 h. Total proteins were extracted, and expression of pSTAT1 (**a**, **d**), total STAT1 (**b**, **e**), PDL1 (**c**, **g**), and pSTAT2 (**f**) were assessed by Western blotting. GAPDH was used as a control for protein loading. Protein signals were quantified and corrected for the corresponding GAPDH value and expressed as fold change compared to untreated cells (CTL) (*n* = 3–4 independent experiments). *p ≤ 0.05, **p ≤ 0.01, ****p ≤ 0.0001 (unpaired Student’s *t* test; significantly different from the control condition at the same time point)

**Fig. 8:**
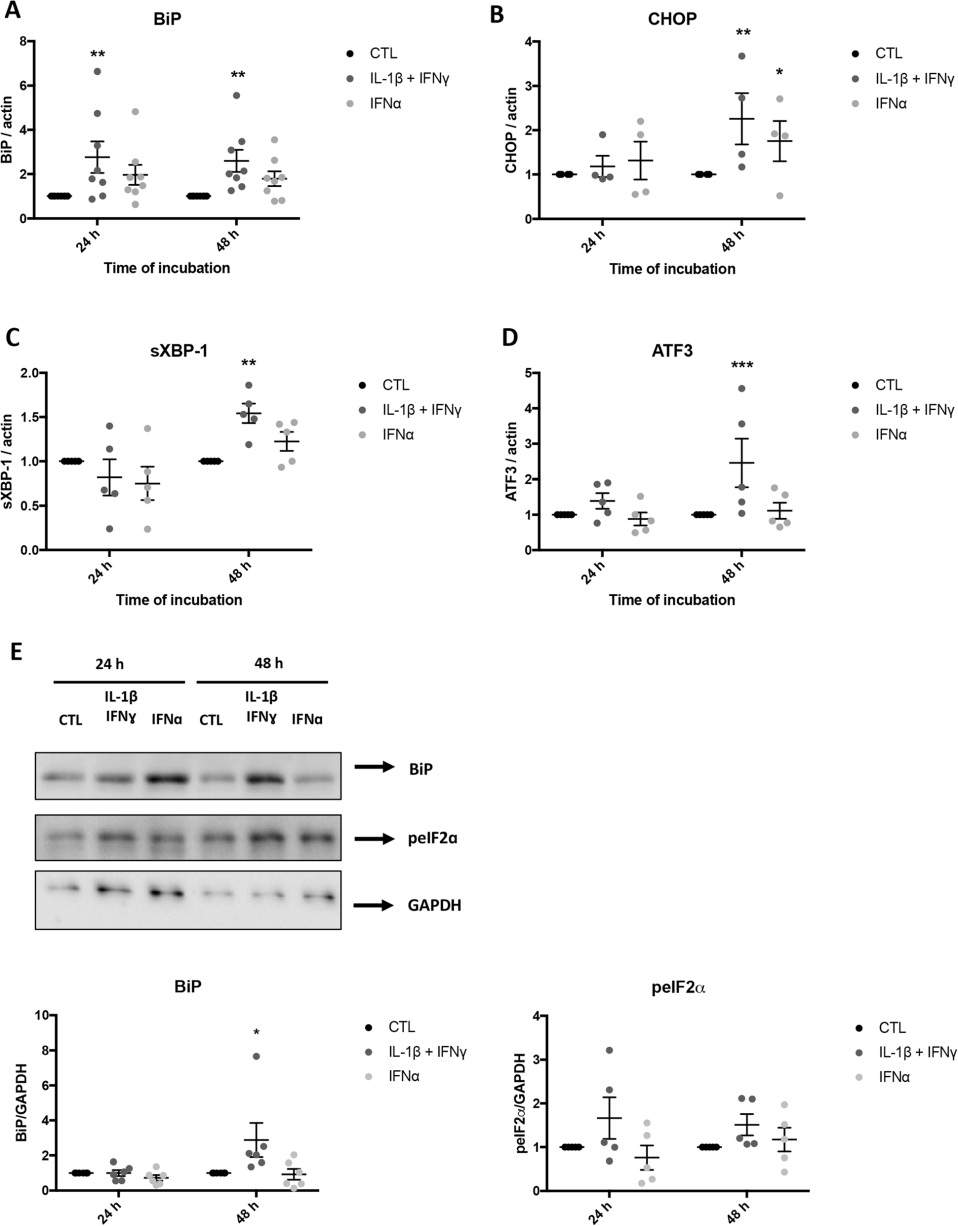
IFNγ + IL-1β or IFNα induce ER stress in iPSC-derived pancreatic endocrine cells. Control iPSCs (HEL115.6) were differentiated into pancreatic endocrine cells and exposed to IFNγ + IL-1β or IFNα. **a**–**d** Total mRNA was extracted and reverse transcribed. The expression of *BiP*, *Chop10*, *sXBP-1*, *ATF3*, and *Actin* (reference gene) mRNAs was quantified using RT-qPCR. Expression was corrected for the corresponding actin value and expressed as fold change compared to untreated cells (CTL) (*n* = 4–8 independent experiments). **e** Total protein was extracted and expression of BiP and phospho-eIF2α assessed by Western blotting. GAPDH was used as a control for protein loading. Representative blots are shown. Protein signals were quantified using Image Studio Lite, corrected for GAPDH, and expressed as fold change compared to the protein expression in untreated cells (CTL) (*n* = 5–6 independent experiments). *p ≤ 0.05, **p ≤ 0.01, ***p ≤ 0.001 (paired Student’s *t* test)

*HLA-ABC* and *CXCL10* are regulated by the transcription factors STAT1/STAT2 [1, 22]. In line with our previous finding using human islets [22], STAT1 phosphorylation was rapidly induced by IFNγ + IL-1β, with maximal induction at 1 h (Fig. 7a). STAT1 phosphorylation was also stimulated by IFNα (1 h) but rapidly decreased after 8 h of stimulation, returning to near basal levels by 24 h (Fig. 7d). IFNα but not IFNγ + IL-1β induced STAT2 phosphorylation, with maximal effect at 1-2 h (Fig. 7f). An increase in total STAT1 and PDL1 expression was observed in response to IFNγ + IL-1β and IFNα, with maximal effect at 48 h (Fig. 7b, c, e, g). Similar effects were observed in human islets exposed to IFNα [22] or to IFNγ + IL-1β [23]. In human islets and EndoC-β1 cells, IFNα activates the transcription factors IRF-1 and IRF-9 [17, 22]. IRF-1 mRNA expression was increased by IFNγ + IL-1β after 24 h of exposure (6.7 ± 2-fold, *P* < 0.05, *n* = 4) but not IRF-9 (1.1 ± 0.3-fold, *n* = 4). On the other hand, IFNα neither induced IRF1 (0.4 ± 1.8-fold) nor IRF-9 (0.4 ± 0.2-fold, *n* = 4) after 24 h of stimulation. Another adult human islet beta cell response to pro-inflammatory cytokines is the induction of ER stress [15, 16]. In iPSC-derived beta cells, IFNγ + IL-1β induced mRNAs encoding the ER stress markers BiP (binding-immunoglobulin protein), CHOP (C/EBP-homologous protein), spliced XBP1 (sXBP1, X-box binding protein 1), and ATF3 (activating transcription factor 3), particularly after 48 h (Fig. 8a–d). IFNα only significantly induced CHOP expression after 48 h (Fig. 8b), but a trend for an increased level of BiP was also present (Fig. 8a). The increase in BiP expression after 48 h of IFNγ + IL-1β exposure was confirmed at the protein level (Fig. 8e). The phosphorylation of the eukaryotic translation initiation factor eIF2α [16, 24] was not significantly changed in iPSC-derived beta cells (Fig. 8e). Comparable results (increase in BiP, CHOP, and ATF3 but not sXBP-1) were obtained with human islets treated with IFNγ + IL-1β or IFNα (Additional file 1: Figures S4D-G and S5D-G).

Finally, we evaluated the possibility to use iPSC-derived beta cells as a model for drug screening. We selected as a proof-of-concept ruxolitinib, a JAK inhibitor previously shown by us to prevent IFNα-induced HLA-ABC overexpression, ER stress, and inflammation in human EndoC-βH1 cells and primary human islets [25]. A 2-h pre-incubation with ruxolitinib nearly completely prevented apoptosis induced by IL-1β + IFNγ or IFNα in islet endocrine cells derived from iPSCs (Additional file 1: Figure S6A-B). Inflammation markers were also reduced, as shown by reduced levels of CXCL10, CCL5, CXCL9, or HLA-ABC in iPSC-derived cells pre-incubated with ruxolitinib (Additional file 1: Figure S6C-J).

## Discussion

We presently evaluated whether pancreatic endocrine cells differentiated from two control iPSC lines using a previously described 7-stage protocol [12] are responsive to the pro-inflammatory cytokines IFNγ, IL-1β, or IFNα. Similarly to adult human islets, these cells are susceptible to IL-1β + IFNγ-induced apoptosis and trigger pro-inflammatory responses (including increased CXCL10 secretion and HLA-ABC expression) [1], dedifferentiation [26], and ER stress [15–17]. IFNα also triggers inflammation and ER stress but also induces apoptosis in pancreatic cells derived from iPSCs, a phenomenon not observed in human islets [17, 22].

We observed that the beta cells derived from iPSCs express receptors for IFNγ, IL-1β, and IFNα, at levels comparable to human islets, and that the combination of IFNγ + IL-1β induces apoptosis already by 24 h of exposure. This phenomenon was confirmed by three different techniques, namely Hoechst 33342/propidium iodide staining, RealTime Glo, and caspase 3 cleavage. These data are in accordance with previous studies suggesting that beta cells differentiated from iPSCs derived from healthy donors or T1D patients are sensitive to a cocktail of pro-inflammation cytokines, i.e., TNFα + IL-1β + IFNγ [13, 14]. Moreover, iPSC-derived beta cells seem to be more sensitive to undergo apoptosis than the other cell types. We also show, for the first time, that the signaling pathways and downstream genes/proteins triggered by IFNγ + IL-1β or IFNα are also similar to the ones observed in human islets [1, 17, 22, 26], with marked STAT1 and/or STAT2 phosphorylation, upregulation of HLA-ABC, of the chemokines CXCL10, CXCL9, and CCL5, as well as markers of ER stress. Of note, the effects of IFNα on STAT1/2 signaling seem to be particularly fast and are rapidly downregulated (after only 4 h), while a more prolonged activation (up to 8–24 h) was observed in primary human islets [22].

A partially dedifferentiated phenotype was seen in iPSC-derived beta cells exposed to IFNγ + IL-1β (but not IFNα), with a decrease in *Ins*, *Gcg*, *Nkx2-2*, and *MafA* expression, but not *Nkx6-1* and *Pdx1*. A partial dedifferentiation was also obtained on a comparable iPSC cell model [13] and in primary human islets [26].

As a whole, human iPSC-derived beta cells reproduce most responses of adult human islets to IFNγ + IL-1β and to IFNα. They thus provide a very useful model to study the pathogenesis of T1D under well-controlled experimental conditions. It is interesting that these iPSC-derived beta cells are not yet fully mature at stage 7 and will only achieve physiological glucose-induced insulin release upon transplantation into immune-deficient mice and after several weeks of in vivo maturation ([8] and our own unpublished data). This suggests that the capacity of beta cells to respond to pro-inflammatory cytokines precedes full functional maturation and is in line with recent observations suggesting that the autoimmune process in T1D may start very early in life, possibly in the prenatal environment [27, 28].

The impact of IFNα on the iPSC-derived beta cells was similar to the human adult beta cell situation, but some differences were detected. Thus, iPSC-derived pancreatic endocrine cells express the IFNα receptor and respond to the cytokine with clear STAT1/2 phosphorylation (present data), similar to adult human islets [17, 22], but they differed in some downstream gene/protein expression. For instance, IFNα induces a marked HLA class I expression and CXCL10 production in human islets [17, 25] but only an increase in HLA class I expression was observed in iPSC-derived islet cells (present data). Furthermore, while IFNα alone does not kill adult human beta cells (apoptosis is only observed when this cytokine is combined with IL-1β) [17], IFNα alone induced apoptosis in iPSC-derived beta cells (present data). These discrepancies may be related to incomplete maturation of some of the early signals downstream of the type I IFN receptor. Indeed, different from adult human islets, IFNα did not induce key downstream transcription factors IRF-1 and IRF-9 (present data).

iPSC-derived islet cells are a valuable disease-in-a-dish model to study inflammatory events in T1D, particularly related to the signal transduction of the pro-inflammatory cytokines IFNγ + IL-1β. These cells present some advantages over primary or clonal human beta cells. First, cells can be generated on-demand from iPSCs, contrary to primary human islets that are much less readily available and are often isolated from older donors. Second, it is possible to generate iPSC from somatic cells obtained from T1D patients, which will allow the study of molecular mechanisms underlying diabetes-associated SNPs (single nucleotide polymorphisms). Finally, these cells represent a valuable tool for the screening for new drugs (as demonstrated for ruxolitinib in the present study) that may protect beta cells against cytokine-induced cell death in early T1D, based for instance on the use of iPSC-derived beta cells obtained from patients with particular polymorphisms that modify cytokine signaling such as TYK2 (tyrosine kinase 2) [29] or PTPN2 (tyrosine-protein phosphatase non-receptor type 2) [30].

## Conclusions

1. iPSC-derived islet cells express receptors for the pro-inflammatory cytokines IL-1β, IFNγ, and IFNα and respond to these cytokines—particularly to IFNγ + IL-1β—similarly to adult human islets, the “golden standard” in the field.
2. iPSC-derived islet cells are a new and valuable disease-in-a-dish model for mechanistic studies on inflammatory events in T1D, particularly related to the signal transduction of the pro-inflammatory cytokines.
3. iPSC-derived islet cells may become also a valuable tool for the screening of new drugs to protect beta cells against cytokine-induced cell death in early T1D.

## Supplementary information

**Additional file 1 Figure S1.** Gene expression across stages of differentiation of iPSCs into pancreatic endocrine cells. **Figure S2.** Expression of IFNγ, IL-1β and IFNα receptors in iPSC-derived pancreatic endocrine cells. **Figure S3.** IFNγ + IL-1β and IFNα induce apoptosis in iPSC-derived pancreatic endocrine cells. **Figure S4.** IFNα does not induce apoptosis but elicits a pro-inflammatory response and ER stress in primary human islets. **Figure S5.** IL-1β + IFNγ induce apoptosis, a pro-inflammatory response and ER stress in primary human islets. **Figure S6.** Ruxolitinib prevents IL-1β + IFNγ- and IFNα-induced apoptosis, inflammation and ER stress in pancreatic endocrine cells derived from iPSCs. **Figure S7.** IL-1β + IFNγ and IFNα do not affect basal insulin secretion in beta cells derived from iPSCs. **Table S1.** RT-qPCR primers. **Table S2.** Antibodies. **Table S3.** Human islet donors presently studied.

## Data Availability

The data supporting the findings of this study are available from the corresponding author, S.D., upon reasonable request.

## Abbreviations

ATF3: Activating transcription factor 3
BiP: Binding-immunoglobulin protein
CCL5: Chemokine (C-C motif) ligand 5
CHOP: C/EBP-homologous protein
CXCL10: C-X-C motif chemokine 10
HLA: Human leukocyte antigen
iPSC: Induced pluripotent stem cell
PTPN2: Tyrosine-protein phosphatase non-receptor type 2
T1D: Type 1 diabetes
TYK2: Tyrosine kinase 2
XBP1: X-box binding protein 1
