# Supplementary material for: Pro-inflammatory cytokines induce cell death, inflammatory responses, and endoplasmic reticulum stress in human iPSC-derived beta cells
Source: Stem Cell Res Ther. 2020 Jan 3;11:7. doi: 10.1186/s13287-019-1523-3 (PMC6942385; doi:10.1186/s13287-019-1523-3)
Supplement: Supplementary file 1 — Additional file 1 Figure S1. Gene expression across stages of differentiation of iPSCs into pancreatic endocrine cells. Figure S2. Expression of IFNγ, IL-1β and IFNα receptors in iPSC-derived pancreatic endocrine cells. Figure S3. IFNγ + IL-1β and IFNα induce apoptosis in iPSC-derived pancreatic endocrine cells. Figure S4. IFNα does not induce apoptosis but elicits a pro-inflammatory response and ER stress in primary human islets. Figure S5. IL-1β + IFNγ induce apoptosis, a pro-inflammatory response and ER stress in primary human islets. Figure S6. Ruxolitinib prevents IL-1β + IFNγ- and IFNα-induced apoptosis, inflammation and ER stress in pancreatic endocrine cells derived from iPSCs. Figure S7. IL-1β + IFNγ and IFNα do not affect basal insulin secretion in beta cells derived from iPSCs. Table S1. RT-qPCR primers. Table S2. Antibodies. Table S3. Human islet donors presently studied. [file 13287_2019_1523_MOESM1_ESM.docx]

**Pro-inflammatory cytokines induce cell death, inflammatory responses and, endoplasmic reticulum stress in human iPSC-derived beta cells**

Stéphane Demine^1,2^, Andrea Alex Schiavo^1^, Sandra Marin-Cañas^1^, Piero Marchetti^3^, Miriam Cnop^1,4^ and Decio L. Eizirik^1,2^

^1^ ULB Center for Diabetes Research, Medical Faculty, Université Libre de Bruxelles (ULB), Route de Lennik 808-CP618, 1070, Brussels, Belgium.

^2^ Indiana Biosciences Research Institute, Indianapolis, Indiana, USA.

^3^ Department of Clinical and Experimental Medicine, University of Pisa, Pisa, Italy.

^4^ Division of Endocrinology, Erasmus Hospital, Université Libre de Bruxelles, 1070 Brussels, Belgium.

**Additional file and methods**

**Human islets**

Human pancreases not suitable for human transplantation were collected as previously reported (1) at the Endocrinology and Metabolism of Organ and Cellular Transplantation Unit of the Cisanello University Hospital in Pisa (Italy). All experiments using human pancreatic samples were performed with the approval of the local Ethics committees of the Pisa University (Italy) and of the ULB (Université Libre de Bruxelles, Belgium). Anonymized organ donor information is provided for the islets used in this study in Supplementary Table 3. Information regarding previously studied human islet donors are available in the corresponding original articles (2, 3).

**Medium insulin accumulation**

Supernatants were collected, and insulin content was determined using a human insulin ELISA kit (Mercodia, Uppsala, Sweden), as described previously [10]. Insulin values were normalised for the protein content (expressed as µg of protein) as determined using a Bradford assay (BioRad, Hercules, CA, USA).

**Figure S1: Gene expression across stages of differentiation of iPSCs into pancreatic endocrine cells.**

Control iPSCs (HEL115.6) were differentiated into pancreatic endocrine cells using a 7-step protocol. At the end of different stages (iPSC=day 0, stage 3, 4, 5, 6 and 7) cells were collected and total mRNA was extracted and reverse transcribed. The expression of *Ins, Gcg, Nkx6-1, Nkx2-2, Pdx1, Ngn3, NeuroD1, Sox9, MafA, Glp1r, SST* and *Actin* (reference gene) was quantified by RT-qPCR. Expression was corrected for actin (n=8-14 independent experiments).


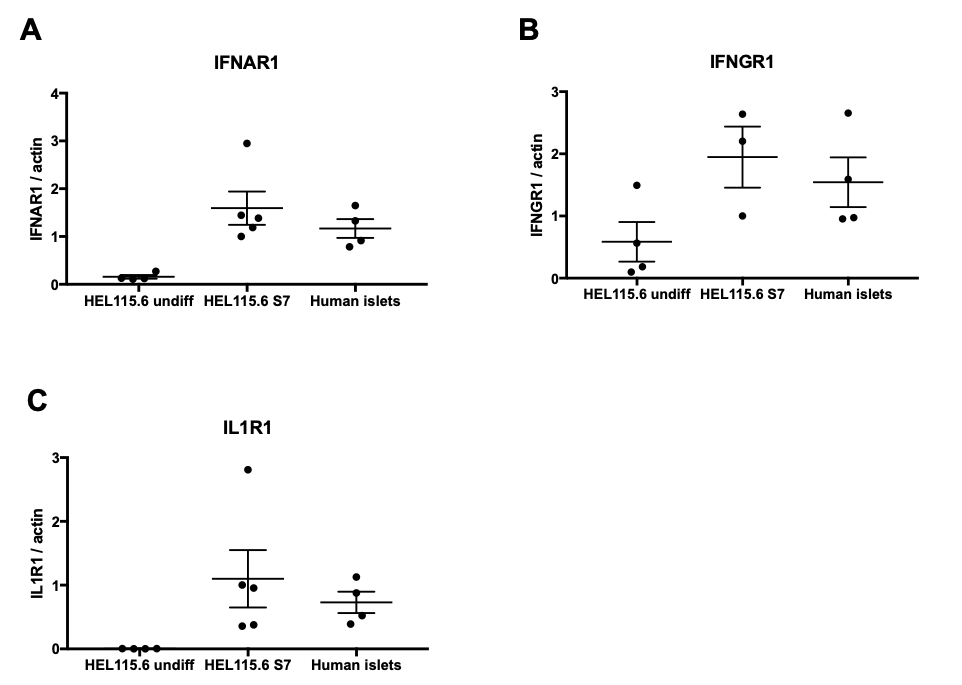


**Figure S2: Expression of IFNγ, IL-1β and IFNα receptors in iPSC-derived pancreatic endocrine cells.**

Control iPSCs (HEL115.6) were differentiated into pancreatic endocrine cells and exposed to cytokines. Undifferentiated HEL115.6 cells and human islets (described in Supplementary Table 3) were used as negative and positive controls, respectively. The expression of *IFNAR1*, *IFNGR1*, *IL1R1*, and *Actin* (reference gene) was quantified using RT-qPCR. Expression was corrected for actin (n=4 independent experiments).


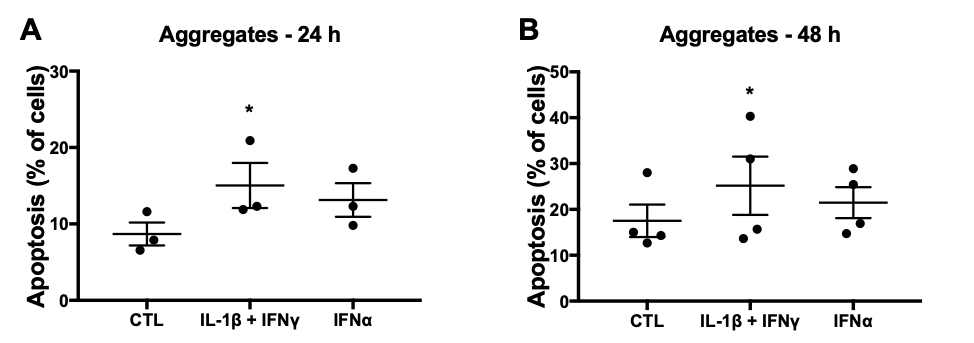


**Figure S3: IFNγ + IL-1β and IFNα induce apoptosis in iPSC-derived pancreatic endocrine cells.**

Control iPSCs (HEL46.11) were differentiated into pancreatic endocrine cells. The aggregates were exposed to IFNγ (1,000 U/mL) + IL-1β (50 U/mL) or to IFNα (2,000 U/mL) for 24 (A) or 48h (B). Apoptosis was measured using Hoechst 33342 and propidium iodide staining and manual counting (n=3-4 independent experiments). **p*≤0.05 (Student’s t-test).

**G**

**F**

**E**

**D**

**C**

**B**

**A**

**Figure S4: IFNα does not induce apoptosis but elicits a pro-inflammatory response and ER stress in primary human islets.**

Primary human islets were dispersed and exposed to IFNα (2,000 U/mL) for 24 (A-G) or 48h (B-G). **A**. Apoptosis was measured after 24 h of incubation using Hoechst 33342/propidium iodide staining and manual counting (n=4 independent experiments). **B-G**. The expression of *HLA-ABC*, *Cxcl10*, *BiP*, *Chop, sXBP-1, Atf3* and *Actin* was quantified using RT-qPCR. Expression was corrected for actin (n=4-5 independent experiments). **p*<0.05, ***p*<0.01, ****p*≤0.001 (paired Student’s t-test (A) or one-way ANOVA followed by unpaired Student’s t-test (B-G)). Data were recalculated from a previously published paper by our group using similar experimental conditions as in the present study (3). Information regarding human islet donors are available in the original article (3).

**G**

**F**

**E**

**D**

**C**

**B**

**A**

**Figure S5: IL-1β+IFNγ induce apoptosis, a pro-inflammatory response and ER stress in primary human islets.**

Primary human islets were dispersed and exposed to IFNγ (1,000 U/mL) + IL-1β (50 U/mL) for 48 h. Apoptosis was measured using Hoechst 33342/propidium iodide staining and manual counting (n=4 independent experiments). The expression of *HLA-ABC*, *Cxcl10*, *BiP*, *Chop, sXBP-1, Atf3* and *Actin* was quantified using RT-qPCR. Expression was corrected for actin (n=5-8 independent experiments). **p*<0.05, ***p*<0.01 (paired Student’s t-test (A) or one-way ANOVA followed by unpaired Student’s t-test (B-G)). Data were recalculated or measured from remaining cDNA from a previously published paper by our group using similar experimental conditions as in the present study (2). Information about human islet donors are available in the original article (2).

**Figure S6: Ruxolitinib prevents IL-1β+IFNγ- and IFNα-induced apoptosis, inflammation and ER stress in pancreatic endocrine cells derived from iPSCs.**

Control iPSCs (HEL115.6) were differentiated into pancreatic endocrine cells, preincubated for 2 h with ruxolinitib (4,000 nmol/L) and exposed to IFNγ (1,000 U/mL) + IL-1β (50 U/mL) or IFNα (2,000 U/mL) for 24 or 48 h. **A-B**. Apoptosis was measured using Hoechst 33342/propidium iodide staining and manual counting (n=3 independent experiments). **C-J**. The expression of *Cxcl10*, *HLA-ABC*, *Cxcl9*, *Ccl5*, and *Actin* mRNAs was quantified using RT-qPCR. Expression was corrected for the corresponding actin value and expressed as fold-change compared to untreated cells (CTL) (n=2-3 independent experiments). *p≤0.05, **p≤0.01 (one-way ANOVA followed by paired Student’s t-test).

**Figure S7: IL-1β+IFNγ and IFNα do not affect basal insulin secretion in beta cells derived from iPSCs.**

Control iPSCs (HEL115.6) were differentiated into pancreatic endocrine cells and exposed to IFNγ (1,000 U/mL) + IL-1β (50 U/mL) or IFNα (2,000 U/mL) for 24 or 48 h. Insulin accumulation in medium was measured by ELISA. Data were normalized for total protein content and expressed as fold-change compared to untreated cells (CTL) (n=8 independent experiments).

| **Target gene** | **Forward primer** | **Reverse primer** |
| --- | --- | --- |
| ***ACTB*** | CTGTACGCCAACACAGTGCT | GCTCAGGAGGAGCAATGATC |
| ***CCL5*** | GCTGCTTTGCCTACATT | CATTTCTTCTCTGGGTTG |
| ***HLA-ABC*** | CAGGAGACACGGAATGTGAA | TTATCTGGATGGTGTGAGAACC |
| ***INS*** | CCAGCCGCAGCCTTTGTGA | CCAGCTCCACCTGCCCCA |
| ***GCG*** | GGGAGAGGGAAGTCATTTGTAA | GTAGAACAGAGCAGGTGAAAAG |
| ***CXCL9*** | GCCATCCTGCCCATAACA | GAGGGCAAGAGCCACAGTAT |
| ***CXCL10*** | GTGGCATTCAAGGAGTAGCTC | GCCTTCGATTCTTGGATTCAG |
| ***sXBP1***  **(spliced)** | CCGCAGCAGGTGCAGG | GAGTCAATACCGCCAGAATCCA |
| ***ATF3*** | TTTGTGTTAACGTGGGAGA | GCTGTCACCACGTGCAGTAT |
| ***PDX1*** | AAAGCTCACGCGTGGAAA | GCCGTGAGATGTACTTGTTGA |
| ***NKX6-1*** | GGGCTCGTTTGGCCTATT | CGTGCTTCTTCCTCCACTT |
| ***SOX9*** | ATCAAGACGGAGCAGCTGAG | GGCTGTAGTGTGGGAGGTTG |
| ***NEUROG3*** | GACGACGCGAAGCTCACCAA | TACAAGCTGTGGTCCGCTAT |
| ***NEUROD1*** | CTATCACTGCTCAGGACCTACT | CCACTCTCGCTGTACGATTT |
| ***NKX2-2*** | GAACCCCTTCTACGACAGCA | ACCGTGCAGGGAGTAACTGAA |
| ***MAFA*** | GCCAGGTGGAGCAGCTGAA | CTTCTCGTATTTCTCCTTGTAC |
| ***GLP1R*** | TCCTGCCACAGACTTGTTCT | GCTGACATTCACGAAGGA |
| ***IL1R1*** | CCGCGCACCGAAGCA | CTTTCATATTCTTCTTGGAGAAGGC |
| ***IFNGR1*** | TAGTTGGTGTAGGCACTGAGGA | GAAGTGACGTAAGGCCGGG |
| ***IFNAR1*** | ATTCCCGACAGACTCATCGC | CGCAGCCGCAGGTGG |
| ***DDIT3***  ***(CHOP)*** | Qiagen, quantitect primer #QT00082278 | |
| ***HSPA5***  ***(BIP)*** | Qiagen, quantitect primer #QT00096404 | |
| ***JAK1*** | CTCACCAGGATGGGGATAAA | AGTTTCCAAGGTAGCCAAGTAT |
| ***IRF1*** | CATTCACACAGGCCGATACA | TGGTCTTTCACCTCCTCGATAT |
| ***IRF9*** | CTCTTCAGAACCGCCTACTTC | GGCTCTCTTCCCAGAAATTCA |
| ***PDL1*** | CCAGTCACCTCTGAACATGAA | ACTTGATGGTCACTGCTTGT |
| ***TYK2*** | TGGCTTGGAAGATGGTGGTG | GTTCCGGCCACACACATTACC |

**Table S1: RT-qPCR primers.**

The table provides the sequences of the RT-qPCR primers, including name of the analyzed gene and the sequences of forward and reverse primers.

| **Target** | **Catalog number** | **Company** | **Application** | **Dilution** | **Incubation time (h)** | **Temperature (°C)** |
| --- | --- | --- | --- | --- | --- | --- |
| **Insulin** | IR002 | Dako (Agilent) | ICC | Ready to use solution | 18 h | 4°C |
| **Glucagon** | G2654 | Sigma | ICC | 1:1000 | 18 h | 4°C |
| **Cleaved caspase-3** | 9661S | Cell Signaling | ICC | 1:400 | 18 h | 4°C |
| **HLA-ABC** | ALX-805-711-C100 | Enzo | ICC | 1:400 | 18 h | 4°C |
| **Pdx1** | 5679S | Cell Signaling | ICC | 1:400 | 18 h | 4°C |
| **Goat anti Guinea Pig**  **Alexa Fluor® 488 conjugate** | A11073 | Thermo Fisher | ICC | 1:500 | 1 h | Room temperature |
| **Goat anti Mouse**  **Alexa Fluor® 546 conjugate** | A11030 |  |  |  | 1 h | Room temperature |
| **Goat anti Rabbit**  **Alexa Fluor® 568 conjugate** | A11036 |  |  |  | 1 h | Room temperature |
| **Goat anti Mouse**  **Alexa Fluor® 647 conjugate** | A32728 |  |  |  | 1 h | Room temperature |
| **Goat anti Rabbit**  **Alexa Fluor® 647 conjugate** | A32733 |  |  |  | 1 h | Room temperature |
| **Phospho STAT1 (Ser701)** | 9171 | Cell Signaling | WB | 1:1000 | 18 h | 4°C |
| **Total STAT1** | Sc-346x | Santa Cruz | WB | 1:1000 | 18 h | 4°C |
| **Phospho STAT2 (Tyr690)** | 4441 | Cell Signaling | WB | 1:1000 | 18 h | 4°C |
| **Total STAT2** | Sc-839 | Santa Cruz | WB | 1:1000 | 18 h | 4°C |
| **HSPA5/ BiP** | 3177S | Cell Signaling | WB | 1:1000 | 18 h | 4°C |
| **Phospho-eIF2ɑ** | 3597 | Cell Signaling | WB | 1:1000 | 18 h | 4°C |
| **PDL1** | ab205921 | Abcam | WB | 1:1000 | 18 h | 4°C |
| **GAPDH** | 2275-PC-100 | Trevigen | WB | 1:1000 | 18 h | 4°C |
| **Horse raddish peroxidase-conjugated anti rabbit** | 711-036-152 | Jackson ImmunoResearch | WB | 1:10000 | 1 h | Room temperature |
| **Horse raddish peroxidase-conjugated anti mouse** | 715-036-150 | Jackson ImmunoResearch | WB | 1:10000 | 1 h | Room temperature |
| **HLA-ABC PE conjugated** | 560964 | BD Biosciences | FC | Ready to use solution | 20 min | 4°C |
| **Insulin Alexa Fluor® 647 conjugate** | 9008 | Cell Signaling | FC | 1:50 | 1 h 30 min | 4°C |
| **Glucagon B421 conjugated** | 565891 | BD Biosciences | FC | 1:50 | 1 h 30 min | 4°C |

**Table S2: Antibodies.**

The table provides the antibodies used in this study, including protein target name, company, catalog number, application, dilution, incubation and temperature time. ICC immunocytochemistry, WB Western blotting, FC Flow cytometry.

| **Age (years)** | **Gender** | **BMI (kg/m^2^)** | **Cause of death** | **Purity (%)** |
| --- | --- | --- | --- | --- |
| 59 | Male | 26.5 | Cerebral hemorrhage | 55 |
| 68 | Male | 27.5 | Cerebral hemorrhage | 42 |
| 49 | Female | 25.4 | Cerebral hemorrhage | 72 |
| 66 | Female | 19.5 | Cerebral hemorrhage | 36 |

**Table S3: Human islet donors presently studied.**

Age, gender, body mass index (BMI) and cause of death of organ donors, and beta cell purity of the human islet preparation (assessed by insulin immunostaining). Islets were kept in culture for 5-6 days before the experiments are performed and were handpicked to increase purity of the preparations.

**References**

1. Marchetti P, Suleiman M, Marselli L. Organ donor pancreases for the study of human islet cell histology and pathophysiology: a precious and valuable resource. Diabetologia. 2018;61(4):770-4.

2. Grieco FA, Schiavo AA, Brozzi F, Juan-Mateu J, Bugliani M, Marchetti P, et al. The microRNAs miR-211-5p and miR-204-5p modulate ER stress in human beta cells. J Mol Endocrinol. 2019. (epub ahead of print; DOI: [10.1530/JME-19-0066](https://doi.org/10.1530/JME-19-0066))

3. Marroqui L, Dos Santos RS, Op de Beeck A, Coomans de Brachène A, Marselli L, Marchetti P, et al. Interferon-α mediates human beta cell HLA class I overexpression, endoplasmic reticulum stress and apoptosis, three hallmarks of early human type 1 diabetes. Diabetologia. 2017;60(4):656-67.
